# Modelling of Three-Dimensional Nanographene

**DOI:** 10.1186/s11671-016-1354-4

**Published:** 2016-03-16

**Authors:** Christos Mathioudakis, Pantelis C. Kelires

**Affiliations:** Research Unit for Nanostructured Materials Systems, Department of Mechanical and Materials Science Engineering, Cyprus University of Technology, P.O. Box 50329, Limassol, 3603 Cyprus

**Keywords:** 3D graphene, Monte Carlo simulations, Tight-binding calculations, Structure, Rigidity, Electronic structure, Conductivity, Absorption

## Abstract

Monte Carlo simulations and tight-binding calculations shed light on the properties of three-dimensional nanographene, a material composed of interlinked, covalently-bonded nanoplatelet graphene units. By constructing realistic model networks of nanographene, we study its structure, mechanical stability, and optoelectronic properties. We find that the material is nanoporous with high specific surface area, in agreement with experimental reports. Its structure is characterized by randomly oriented and curved nanoplatelet units which retain a high degree of graphene order. The material exhibits good mechanical stability with a formation energy of only ∼0.3 eV/atom compared to two-dimensional graphene. It has high electrical conductivity and optical absorption, with values approaching those of graphene.

## Background

Recent efforts in graphene research include the realization of three-dimensional (3D) bulk materials with graphene nanoplatelets (nanoribbons) as their building blocks [1–4]. These architectures are envisioned such as to retain the exceptional properties of 2D graphene in addition to providing mechanical robustness, high surface area, and macroporosity, properties that are invaluable for a plethora of applications including catalysis, sensors, and energy storage and conversion (carbon-based supercapacitors, electrodes in Li-ion batteries, active materials in solar cells), among others. In addition, the preservation of high conductivity and electron mobility is essential for any possible electronic applications.

The reported up-to-date 3D graphene structures, produced by a variety of experimental techniques, such as chemical vapor deposition (CVD) [1], assembly, and chemical treatment of graphene oxide (GO) sheets [5, 6], or pyrolysis and etching of sol-gel organic polymers [4, 7], generally visualize the resulting 3D networks as porous and spongy, composed of nanometer-sized (2–10 nm) curved nanoplatelets randomly oriented, interconnected, and intertwined. The aim is to avoid restacking of individual sheets which suppresses conductivity and mechanical strength and lowers the surface area. However, besides their porous nature, the 3D structures are reported to be distorted, in the sense that the nanoplatelets are deformed, possibly containing both topological and point defects. Also, at the junction areas where the platelets meet and merge, and at the edges, one might expect carbon hybridizations other than the planar sp ^2^ geometries of the graphene plane, which may possibly alter the anticipated electronic conduction and optical properties of these materials.

This inherent disorder, therefore, raises a number of questions about the stability and the microstructure of these porous networks, and how this disorder influences the optoelectronic properties of 3D nanographene. Some key issues to be tackled include the rigidity and robustness of the networks, their high specific surface area, whether and in what degree they preserve graphene order and bonding percolation, and if conduction and optical absorption are strongly influenced by the deformations of the nanoplatelets.

Here, we report what is presumably the first attempt to simulate and study theoretically the structural, mechanical, and optoelectronic properties of realistic 3D nanographene (3D-NG) materials. We achieve this through a combination of atomistic Monte Carlo simulations, to generate the networks in a random unbiased way, and tight-binding calculations to relax them properly and extract their basic properties. The outstanding finding of our studies is that 3D-NG exhibits good mechanical stability and high electrical conductivity and optical absorption, approaching those of 2D graphene.

## Methods

### Monte Carlo simulations

We construct and characterize the 3D-NG networks in two steps, following a procedure we used earlier to construct carbon nanofoam networks [8]. In the first step, we generate networks in a random and unbiased way using atomistic Monte Carlo (MC) simulations in the (N,P,T) isothermal-isobaric statistical ensemble [9, 10]. The resulting networks are generic, representing materials produced by the various methods mentioned above. The networks are formed by condensing a “vapor” containing various randomly oriented nanoribbon graphene units with lengths 2–5 nm, both of the zig-zag and armchair types, initially positioned at large distances, under small external pressure and at 1000 K. The energetics are described by the Tersoff empirical potential [11]. We use cubic supercells of ∼1300 carbon atoms, with periodic boundary conditions, thus simulating the bulk of the material. During condensation, the nanoribbon units perform rotational moves while approaching each other. Thus, when they interact with neighboring units, they randomly agglomerate and interlink, forming a covalently bonded network, with a certain degree of deformation and fragmentation inducing disorder around the junctions and curvature alterations on the nanoribbon surfaces. The resulting networks are then relaxed at 300 K.

### Tight-binding calculations

In the second step of our procedure, the 3D-NG networks generated by the MC method are fully relaxed using tight-binding molecular dynamics (TBMD) simulations in the (N,V,T) canonical ensemble. We first anneal extensively the networks at 2000 K in order to bring the structures out of any local energy minima, and then we relax the volume/density at 300 K, where the structural, mechanical, and electronic properties are inferred. The calculations are carried out within the TB framework developed at the Naval Research Laboratory (NRL) [12]. This is a two-center non-orthogonal model, using *s* and *p* atomic-like orbitals and distance- and environment-dependent parameters for transferability between different structures. The successful description of various carbon phases by this model was previously demonstrated [8, 13]. Due to the large size of the supercell (thus small Brillouin zone), we only use the *Γ* point for the calculation of energies and optoelectronic quantities.

**Fig. 1:**
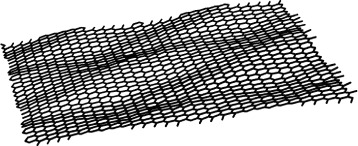
Undulations and rippling of the graphene layer at 300 K, as reproduced by TBMD simulations using the NRL hamiltonian. The height of the ripples are of the order of ∼1 Å

The model also describes nicely the well-known [14, 15] instability of the ideal planar graphene layer towards roughening, characterized by undulations and ripples. Figure 1 shows a snapshot of the graphene plane from TBMD simulations at 300 K. Ripples of the order of ∼1 Å are evident.

## Results and discussion

### Microstructure

**Fig. 2:**
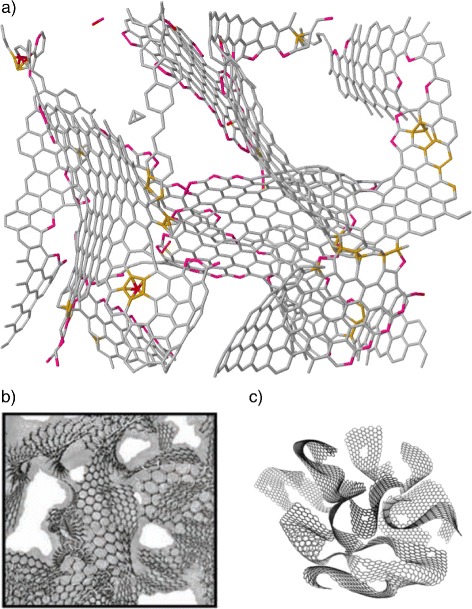
**a** Model of a 3D-NG network, with periodic boundary conditions, composed of curved graphene nanoplatelets. The density is 0.5 g/cm ^3^. Pore size is of the order of 1–2 nm. Grey, orange, and magenta denote sp ^2^, sp ^3^, and sp ^1^ bonding, respectively. **b** Experimental model of 3D-NG derived from a polymer-based top-down approach [4]. **c** Experimental model of 3D-NG derived by assembly of GO sheets [6]

We first begin with the microstructure of 3D-NG. Panel (a) of Fig. 2 shows a representative covalently bonded 3D network generated with the procedure described above. Its nanoporous nature is evident, with a pore size of ∼1–2 nm. The main characteristic of this structure is that the randomly interacting and interlinked units do not remain intact, preserving their shape and planarity, but they are deformed, curved, and sometimes fragmented, which results in the appearance of non-sp ^2^ atoms and the formation of new, both large and small, atomic rings. These have a profound effect on the electronic properties, as shown below. The density of the network is 0.5 g/cm ^3^, making the material mechanically very robust. This should be considered as an upper limit to the density of 3D-NG, in view of the rather small size of the computational box used here and the possibility of larger pore sizes that inevitably lower the density. Biener et al. [4] reported a similar density of 0.2 g/cm ^3^ for 3D-NG prepared by pyrolysis and etching of sol-gel organic polymers. Ultra-low density (of the order of mg/cm ^3^) foamy structures might also be possible to achieve, but these should be less stable.

Our structure compares favorably with experimental models of 3D-NG, extracted from samples prepared by polymer pyrolysis [4] and assembly of GO sheets [6], and shown in panels (b) and (c) of Fig. 2, respectively. Both theory and experiment show that these 3D networks, although porous and with deformed graphene units, exhibit good bonding percolation which should be significant for conduction.

As expected, the main bonding hybridization in our model in Fig. 2a is sp ^2^, the fraction of such sites being 88 %. However, there is also a small fraction (3 %) of sp ^3^ sites, some of them located at the junctions between the units as linking geometries, while the rest (9 %) are sp ^1^ sites, found both at the junctions and decorating the internal pore surfaces. The curved nature of the nanoplatelets is reflected in the appearance of both large and small atomic rings, other than the dominant (70 %) six-membered (6 m) rings forming the ideal graphene layer. We find, for example, a large fraction of 9-m rings (9 %), 7-m rings (5 %), and 8-m rings (4 %). There are also small 3-m rings (5 %) and 5-m rings (8 %). Non-6-m rings and non-sp ^2^ sites put their signature on the electronic density of states to be discussed below.

We calculate the specific surface area (SSA) of our networks to be in the range of 3000–3200 m ^2^/g, compared to 2636 m ^2^/g for ideal monolayer graphene. Our values are in accord with the experimental value of 3000 m ^2^/g reported by Biener et al. [4]. Most likely, the excess SSA compared to ideal graphene is contributed by the internal pore surfaces. Thus, the smaller the nanoplatelet units, the larger the SSA is expected to be. This feature makes 3D-NG invaluable for catalysis and energy storage applications.

**Fig. 3:**
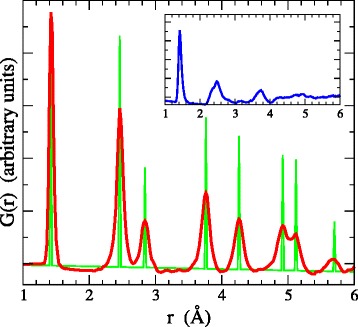
Reduced radial distribution function G(r) of 3D-NG (*red curve*) compared to the G(r) of the graphene plane (*green curve*). *Inset* (*blue curve*) shows the G(r) of a low-density a-C network

Figure 3 shows the reduced radial distribution function G(r) of the 3D-NG network visualized in Fig. 2a, compared to the G(r) of the graphene plane and the G(r) of a low-density (1.6 g/cm ^3^) amorphous carbon (a-C) network (shown in the inset), which was generated with the same TBMD methodology. There are some striking differences between the G(r) of 3D-NG and a-C. One comes from the feature between the second and third peak, at ∼2.8 Å. This extra peak corresponds to the third-nearest-neighbor distance in the graphene hexagon, and is absent in a-C. Another notable difference is that peaks beyond remain intense in 3D-NG, while they vanish in a-C. These differences show that not only short-range order but also medium-range order correlations, and thus graphene order, are highly preserved in 3D-NG despite its distorted nature. It is evident that the sharp peaks in the G(r) of graphene are broadened into the peaks of 3D-NG at exactly the same radial distance. Overall, we may characterize 3D-NG as a continuous random network (CRN) at the microlevel, in the sense that the units percolate throughout but they are randomly oriented, while high order prevails at the nanolevel.

### Mechanical stability

**Fig. 4:**
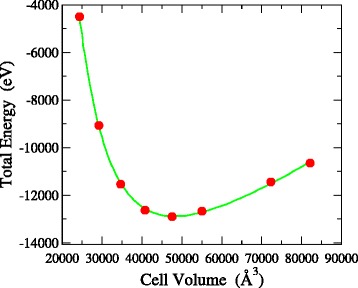
Total energy versus volume curve of a 3D-NG network. The curve is fitted to the Murnaghan’s equation of state to yield the bulk modulus of the material

As a stringest test of the mechanical stability of 3D-NG, we calculated the bulk modulus *B*_0_ of our networks by fitting their energy versus volume curves to the Murnaghan’s equation of state [16]. Such a fit is shown in Fig. 4. The fit is excellent, yielding a *B*_0_ of 60 GPa. All networks studied have values of *B*_0_ in the range of 55–65 GPa despite their porous nature. This is lower than values for typical 3D carbon materials with low density, such as graphite-like a-C (*B*_0_∼200–250 GPa), but still high enough to make 3D-NG durable for potential applications. Again, our values should be an upper limit since for networks with larger pores and smaller density we expect lower moduli. Work is in progress to estimate the hardness of the material.

Further evidence for the stability of 3D-NG networks is provided by calculating their formation energies relative to the undulated graphene plane at 300 K. We find values ranging from 0.2 to 0.5 eV/atom. For example, the total energy of the network shown in Fig. 2a is calculated to be −9.94 eV/atom, to be compared to −10.25 eV/atom for the graphene plane, yielding a formation energy of ∼0.3 eV/atom. Thus, 3D-NG is energetically stable and feasible in the bulk form studied here, representing thin films deposited with good adhesion on appropriate substrates.

### Electronic structure

**Fig. 5:**
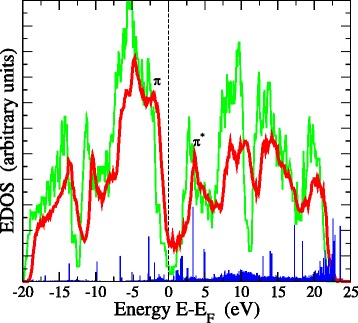
EDOS of 3D-NG (*red curve*) compared to the EDOS of monolayer graphene. *Vertical spikes* (*blue*) indicate a typical distribution of localized states from IPR analysis

We now proceed to the study of the electronic structure of 3D-NG. Figure 5 plots the calculated TB electronic density of states (EDOS) of the 3D-NG network portrayed in Fig. 2a compared to the EDOS of graphene, both averaged over several steps at 300 K. The EDOS of graphene verifies its semimetallic nature, the few states at the Fermi level *ε*_*F*_ arising from the disorder due to the ripples. The EDOS of 3D-NG, on the other hand, follows more or less the shape of the graphene EDOS, which is in accord with the experimental work by Biener et al. [4], but exhibits considerably more states in the region around *ε*_*F*_, it is completely gapless. As these states around *ε*_*F*_ are *π* orbital states, and given the deformations induced by crosslinking, one wonders whether their *π* orbitals are properly aligned so as to keep their delocalized nature, as in graphene, and give a metallic character to the material.

We investigate this issue by calculating the inverse participation ratio (IPR) defined by $P = \sum _{i} {c_{i}^{4}}$, where *c*_*i*_ are the coefficients in the expansion of the eigenstates in terms of the local orbitals, and which is a measure of the localization of the electronic states in the system. The localized states and their IPR are denoted by vertical spikes in Fig. 5. This analysis shows that the *ε*_*F*_ region is practically free of localized states. This is in sharp contrast to a-C where the *π* and *π*^∗^ states near *ε*_*F*_ are localized, due to misalignment of the *π* orbitals, causing the low conductivity of the material [17]. The IPR analysis shows that there are localized states, but these lie deeper in the valence and conduction bands. The major component to localization originates from sp ^1^-bonded atoms at the internal surfaces, while sp ^2^ and sp ^3^ sites contribute minimally.

The proper alignment of *π* orbitals in the presence of deformations can be explained by noting that the large-membered rings in the network induce locally a planar geometry, causing the proper alignment of *π* orbitals. This is significant because many of the atoms contributing significantly at *ε*_*F*_ are sp ^2^-bonded atoms located at large 8- and 9-m rings. Otherwise, such atoms would contribute localized states at *ε*_*F*_, as defect atoms do. We observed a similar phenomenon of large-ring-induced local planarity in our previous studies [8] of carbon nanofoams composed of schwarzite units.

### Optoelectronic properties

We now proceed to the calculation of the optoelectronic properties of the material. We first examine the electrical conductivity. Our finding that the *π* states in 3D-NG are delocalized at *ε*_*F*_ is expected to have a strong effect on the conductivity. In its standard frequency-dependent form, this is given by

(1) $$ {}\sigma(\omega) = \left(\frac{2 \pi e^{2}}{3 V \hbar^{2} \omega} \right) \sum_{i,f} (E_{f}-E_{i})^{2} |\langle f|\textbf{r}|i \rangle|^{2} \delta(E_{f}-E_{i}-\hbar\omega),   $$

where *E*_*i*_ and *E*_*f*_ are the energies of the initial occupied valence eigenstates |*i*〉 and the final unoccupied conduction eigenstates |*f*〉, respectively. The rigorous relation $\hbar \langle f|\textbf {P}|i \rangle = im (E_{f}-E_{i}) \langle f|\textbf {r}|i \rangle $ [18] has been utilized to express the momentum matrix elements in terms of the position matrix elements, which are readily calculable. To calculate them, we make use of the LCAO (linear combination of atomic orbitals) expansion of the |*i*〉 and |*f*〉 eigenstates into atomic states (orbitals) |*a*〉 and |*b*〉

(2) $$ |i\rangle = \sum_{j,a} c_{j,a}^{(i)} |a\rangle\qquad\qquad |f\rangle = \sum_{j,b} c_{j,b}^{(f)} |b\rangle,   $$

where $c_{j,a}^{(i)}$ and $c_{j,b}^{(f)}$ are the expansion coefficients (eigenvector components), and the summations run over all atomic sites in the unit cell *j* and all atomic orbitals in the basis set. The basis sets of the atomic orbitals |*a*〉 and |*b*〉 are in principle different. Considering the LCAO expansions in Eq. (2), the position matrix elements are expressed as a summation over the expansion coefficients

(3) $$ \langle f|\textbf{r}|i \rangle = \sum_{j,l} c_{j,l}^{(f)*} c_{j,l}^{(i)} \langle l|\textbf{r}|l \rangle = \sum_{j,l} c_{j,l}^{(f)*} c_{j,l}^{(i)} \vec{r}_{j},  $$

where the index *l* runs over both initial and final states, and taking into account that only the diagonal elements of the position operator survive (〈*k*|**r**|*l*〉=0) [18]. Using this expansion greatly facilitates the calculation of conductivity through Eq. (1). The direct current (DC) conductivity is obtained in the limit *ω* (or *E*) → 0.

**Fig. 6:**
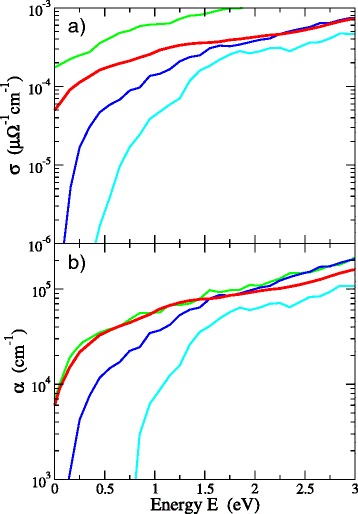
**a** Conductivity of 3D-NG (*red curve*) compared to that of a graphene layer (*green curve*), of a low-density a-C cell (*blue curve*), and of a tetrahedral a-C cell (*light blue curve*). **b** Optical absorption of the above materials

The results of our calculations of the conductivity as a function of the energy are given in Fig. 6a. Let us first point out that the calculated (with the present TB hamiltonian) DC conductivity *σ*_*DC*_ of the graphene layer is ∼ 2×10^−4^*μΩ*^−1^*cm*^−1^. This agrees with several experimental measurements in graphene materials [19–21]. For example, the work in Ref. [21] on graphene sheets measured a value of 2×10^−3^*μΩ*^−1^*cm*^−1^. This agreement provides us with a benchmark and shows the reliability of our approach for the calculation of the transition matrix elements. For 3D-NG, our calculations predict that *σ*_*DC*_ is of the order of ∼ 5×10^−5^*μΩ*^−1^*cm*^−1^, which underlines that 3D nanographene retains in a large extent the conductivity of 2D graphene. For comparison, the experimental work of Worsley et al. [7] on 3D-NG reported a lower value of ∼ 10^−6^*μΩ*^−1^*cm*^−1^, while Chen et al. [1] reported a value ∼ 10^−5^*μΩ*^−1^*cm*^−1^, which is closer to ours. Obviously, the conductivity of covalently bonded 3D-NG will depend on the connectivity and percolation of units, the pore size, and the density.

Note that, as shown in Fig. 6a, the conductivity of 3D-NG is 3–4 orders of magnitude higher than the well-established values of *σ*_*DC*_ of graphite-like a-C, which is highly sp ^2^-rich, and more than eight orders of magnitude higher than sp ^3^-rich tetrahedral a-C [22]. Our computed values are ∼ 10^−8^*μΩ*^−1^*cm*^−1^ for the former and practically zero for the latter. As discussed above, this is due to the strong localization of *π* states in the a-C phase due to the misalignment of *π* orbitals.

The 3D-NG materials are also highly absorbtive, as demonstrated in Fig. 6b. The absorption coefficient of various networks, given by $\alpha (\omega)=\frac {\omega \epsilon _{2}(\omega)}{nc}$, where *ε*_2_(*ω*) is the imaginary part of the dielectric function and *n* is the refractive index of the material [23], reveals that their absorbtance is similar to a graphene layer and considerably higher than that of low-density a-C and, especially, ta-C which has a much sharper absorption edge. The strong absorption makes 3D-NG promising materials for optical applications.

## Conclusions

In conclusion, we reported here the first simulational and theoretical study of the structural, mechanical, and optoelectronic properties of realistic 3D nanographene materials, through Monte Carlo simulations and tight-binding calculations. We found that 3D-NG is characterized by nanoporosity and high specific surface area. It is a random network at the macrolevel but with high graphene order at the nanolevel, within the nanoplatelet units. It exhibits good mechanical stability and high electrical conductivity and optical absorption, approaching those of 2D graphene. These characteristics and the calculated relevant quantities are in good agreement with experimental measurements. Actually, some of them, such as the electrical conductivity, are even higher than what is reported experimentally. This may indicate that there is room for improvement of these materials at the lab through better unit connectivity and percolation, and pore size and density control.
